# Micro-Organisms in Dental Caries

**Published:** 1900-07

**Authors:** Kenneth W. Goadby

**Affiliations:** L.D.S. (Eng.)


### Abstracts and Translations.

### MICRO-ORGANISMS IN DENTAL CARIES.[1]

BY KENNETH W. GOADBY, L.D.S. (ENG.).[2]

CONSIDERABLE attention has been directed to the study of the micro-organisms of the human mouth during the last few years, and the literature on the subject is rapidly accumulating, so that within the narrow limits of the present paper it is impossible to even refer to some of the interesting problems with which the whole subject teems. I have, therefore, selected one especial issue which is of interest to those engaged in the practice of dental surgery.

But before discussing the bacteriology of dental caries, let me draw your attention for a few moments to one or two general considerations of bacteriological work, and note in passing some of the special difficulties attendant on the study of the flora of the mouth.

The mouth, as you are all aware, is admirably adapted to the growth of bacteria, and for this reason, as well as its extreme liability to infection, becomes the resting-place of the many varieties of organisms found both in air, water, and foods; but, as has been pointed out by Sanarelli, Miller, Washbourn, and myself, many of the species quickly disappear, due either to the reaction of the medium in which they find themselves, the temperature, or the action of other organisms. To this latter condition I shall have occasion to refer later.

We are thus met at the commencement with an almost illimitable number of organisms which may occur in the buccal cavity, as well as another equally difficult problem,—*i.e.*, the media upon which to cultivate, many of the buccal inhabitants refusing to grow upon the ordinary laboratory media. There are many other conditions, some of which I have noted elsewhere.[3] It will be seen, therefore, that to fully understand what are proper mouth bac-

---

[1] Read at the Annual General Meeting of the British Dental Association at Ipswich, May, 1899. A grant in aid of this work was made by the British Medical Association.

[2] Demonstrator of Practical Dentistry, Guy's Hospital.

[3] Journal of the British Dental Association, October, 1898, to March, 1899. Trans. Odont., June, 1898.

teria we must meet the difficulty by careful examinations of large numbers of mouths and noting the bacteria most commonly present, whilst the question of media can only be solved by constant and arduous work.

But let us for a time consider the first of these two special problems. Evidently if the various workers in the field are to contribute to the common progress of the subject, the groundwork of their methods must be upon the same plan; that is to say, in all descriptions [1] standard media and processes must be adopted, as well as the special methods which must always be clearly stated, whence it follows that the merely accidental varieties will be separated from those bearing a proper relation to the pathological conditions in which so much remains to be elucidated in the domain of dental surgery.

But what constitutes a new organism? Certainly not morphological differences alone, as so many observers have relied upon (Miller, Vincentini, etc.). No; a new organism must present some points in its biological, pathological, biochemical, as well as its morphological conditions, by which it may be recognized, and in virtue of which it differs from other known organisms. With these reactions carefully worked out upon standard media the newcomer obtains a scientific status; with these it can be recognized by another observer, and the personal equation eliminated by individual work.

In no other region of the domain of bacteriology is morphology more elusive and untrustworthy than among the organisms of the mouth: the curious greasy nature of "Leeuwenhoek's Materia Alba," the albumen in the saliva, the *débris* of food and epithelial cells, and, more important still, the pleomorphism of the organisms themselves, all combine to render the morphological forms taken direct from the mouth extremely slender evidence, *per se,* upon which to base conclusion or theory.

In the April number of the *Dental Cosmos* Leon Williams gives photographs of a number of morphological forms stained from the mouth direct, and has to a certain extent confirmed some of Vincentini's work, in so far as he shows that there is ground for some of the statements in Vincentini's book, although not binding himself to adopt all Vincentini claims for the leptothrix racemosa.

---

[1] Committee of American Bacteriologists, Report of Committee, 1898.

I am able, with much pleasure, to confirm a certain amount of the statements, but by no means all, whilst in the first place the method of staining adopted by Leon Williams appears to me rather crude for bacteriological work (staining *débris* in gentian violet and glycerin, in a watch-glass,—*Dental Cosmos,* April, 1899).

Again, the term "diphtheroid organism," as applied by Williams to certain morphological forms, which—in the photographs, at any rate—no more resemble the diphtheria bacillus than any other slightly curved bacillus, is a term much to be regretted, especially so as other well-known organisms morphologically resembling the Klebs-Loeffler bacillus are often to be found in the mouth (*cf.* Hofmann), to mention only one variety.

Further, the chance arrangement of bacilli in clumps such as photographed by him is not in the least typical of diphtheria, although it might have been considered so ten years ago.

And, again, involution forms are of such common occurrence in any cultivation of bacteria (*cf.* streptococcus, anthrax, cholera, subtilis, mesentericus, etc.), that much care must be exercised in giving the true morphology of any bacilli. It is also much to be regretted that the experiments as to acid production should have been conducted with such well-known producers of acid as the two staphyloccocci and sarcina lutea, as reference to any good textbook on bacteriology would have given the information (*cf.* Sternberg, "Manual of Bacteriology").

The acid production by organisms is a curious phenomenon, more particularly as the natural growth of bacteria results in an alkaline reaction, and that only certain species in the presence of proper media can bring about a reversion of the alkaline reaction.

It is, therefore, a grave error to mass into one catalogue all organisms which occur upon the teeth as acid producers, although many of them are; and Leon Williams's conclusion, "this complete demonstration of acid-producing forms," to quote his own words, is in no wise a "complete demonstration" until the organisms have all been cultivated on artificial media and their individual efforts studied, a matter Williams tacitly admits he has not done.

An organism Williams has overlooked, or has not mentioned, is one that Washbourn and myself have constantly pointed out to occur in every mouth, and I find that on referring to my notebooks I have observed the streptococcus in three hundred different mouths, and in one hundred and fifty I have isolated them in pure cultivation.

I have experienced no difficulty whatever in obtaining specimens of the fruitful heads by staining by the ordinary Gram method, and decolorizing with chloroform and alcohol, making a careful emulsion of the material used first in distilled water, and allowing the drop to evaporate on the coverslip. These "fruitful heads" consist, as Leon Williams has pointed out, of a mass of cocci-like bodies arranged around the whole length, at the end of a thread. The size is large, the threads being one and one-half to two millimetres wide, the thicker portions of the head three to five millimetres wide; there are also arranged around these, but quite detached, the circular masses photographed by Williams, and termed transverse sections; why, it is not quite clear. I shall have occasion to refer to these again.

These curious threads, with cocci-like bodies arranged round them, can be easily studied in the hanging drop direct,—a method I am surprised has not been adopted by Williams, as it is one of the routine methods adapted in bacteriological work.

I must confess that I was extremely sceptical at first as to the real existence of these fruitful heads, especially as the agglomeration of cocci about a thread may easily be produced by growing a freely growing coccus like staphylococcus aureus or albus in a broth culture of B. subtilis.

Small plates [1] were then examined under the microscope, and the existence of the curious forms noted by Williams and Vincentini were plainly to be seen. The coverslip plates were then incubated at 40° C. in the case of the agar and broth, and examined at each subsequent period of twelve hours for six days, and the results compared with one another. Subsequently an agar coverslip plate was made in the same way, and a group of heads brought into the field, and then the microscope placed in the hot incubator at 40° C., the preparation being examined at intervals of twelve hours, and camera lucida drawings made at each observation to check the development.

Several curious facts were observed. (1) There was little alteration in the general conditions for the first forty-eight hours,

---

[1] These plates were made by smearing agar or gelatin over coverslips, the plates being cemented with Canada balsam to hanging drop-rings after inoculation.

after which time short thread-like forms were observed growing from some of the cocci-like bodies of the head. (2) In broth, the heads, in forty-eight hours, became in many instances surrounded with masses of cocci; in others, the threads were growing from the spores, but the masses of cocci did not develop into threads. The spores showed considerable Brownian movement, but were not motile.

The threads themselves showed several curious points. (1) Some of them degenerated to a considerable extent, and showed, in their interior, segmentation of the protoplasm into cocci-like bodies. Some of them lay outside the threads. (2) In agar and broth specimens chains of streptococci were seen growing from the cocci on the threads; on the agar plates, genuine streptococcal colonies were found attached to the threads, and which gave cultures of the ordinary mouth streptococcus upon sub-cultures being made. In the threads which showed the attachment of streptococci a considerable erosion of the thread was observed. The cocci were easily differentiated from the spore-like bodies. (3) The short bacilli and spirilla had no apparent connection with the threads, and were moving freely; on agar the bacilli formed colonies. (4) Some of the threads showed branching and sporulation of the penicilium type. (5) Eventually some of the threads underwent formation into cocci-like bodies, by segmentation of the protoplasm, through a large portion of their length. (6) The small clusters of spores (Williams's transverse section) showed development of branches radiating from the central mass. (7) Some threads showed pseudo-branching. The threads are by no means all concerned in this formation of spores, and many of them belong to other species, as will be seen later.

It is somewhat interesting to note that the thread-forms around which the streptococcus develops, and whose contour shows erosion, appear not to develop spores, so that the streptococcus would appear to be antagonistic to its growth, more particularly as on most media the streptococcus "grows down" most other organisms, as has been pointed out by Washbourn [1] and myself.

One point in particular is brought out by the foregoing experiments,—*i.e.*, that the process of development actually watched is, (1) spore, (2) thread from spore, (3) spore. Nothing approach-

[1] Trans. Odont. Soc., June, 1896.

ing a sexual method of reproduction was noticed. There is, however, no evidence to show what part, if any, this interesting organism plays in dental caries, and, until it has been isolated and its biological characters studied, the matter is entirely conjecture.

The term "leptothrix racemosa" is one that may remain for the present, but the organism much more resembles the crenothrix of Rabenhorst than anything else (see *résumé*).

Let us now turn our attention more closely to dental caries. The subject naturally falls under two heads, as far as the bacteriology is concerned: (1) the organisms occurring upon the surface of the decaying dentine; (2) the organisms found in the deeper layers.

Naturally, any organism found in the mouth may obtain access to the carious tooth, but among these organisms certain ones are constantly to be found, many of them being acid producers, others liquefiers of gelatin. The first outcome of investigation of the organisms occurring upon carious dentine showed that a far larger variety and quantity of organisms exists upon the surface than in the deeper layers of necrosed tissue.

The next point appears to be the somewhat curious fact that the organisms which are surface growers,—*i.e.*, aërobic,—are in many instances liquefiers of gelatin; they may or may not produce acid; many of them do not change the color of litmus milk. Most of the species I have isolated from the surface of carious dentine are aërobic liquefiers, some of them liquefying the gelatin with great rapidity, and also liquefying coagulated blood-serum. A small fraction are anaërobic liquefiers, among these latter being B. mesentericus (ruber, vulgatus, fuscus). Many of these organisms found upon the surface have the power of producing a brown discoloration of the media in which they are cultivated, especially upon glycerin agar and potato.

The organisms which I have isolated from the deep layers of caries are naturally anaërobic, though facultative aërobic, and produce acid rapidly when grown in litmus milk or glucose media; they do not, as a rule, liquefy gelatin, and do not discolor the medium.

Miller's work on the destruction of the tooth substance by bacteria is so well known to you that I have not considered it necessary to repeat his experiments in this direction. However, one point required clearing up, as Miller experimented with impure cultivations, and his results were general. He has well shown that the process of caries is always preceded by a destruction of the calcium salts by the acids produced by the organisms, a matter that my experiments confirm, as will be seen later.

I thought, however, that it would be interesting to determine the specific action of liquefying organisms upon dentine, for it must not be supposed that all organisms capable of liquefying nutrient gelatin also possess the power of digesting the decalcified tooth cartilage or chondrinogen, a point well brought out in the following experiments.

Slices of healthy teeth were decalcified in ten per cent. hydrochloric acid in distilled water, and, when entirely decalcified, were washed in ten per cent. solution of ammonium hydrate, subsequently in sterilized water.

The slices were then placed upon the surface of nutrient media, and the tube inoculated with a pure cultivation of a liquefying organism. Uninoculated control tubes were also made.

Three organisms were employed,—(1) B. furvus (Goadby)[1] liquefies blood-serum and gelatin. (2) B. mesentericus fuscus liquefies blood-serum and gelatin. (3) B. plexiformis (Goadby) liquefies gelatin, blood-serum not affected. All of them isolated from the surface of carious dentine. The media used were agar, blood-serum, gelatin (agar and gelatin three cubic centimetres normal NaOH per litre alkalinity).

In forty-eight hours in (1) and (2) upon blood-serum the slice of dentine was considerably softened. In four days the slice was entirely liquefied. The reaction of the medium was strongly alkaline. With the third organism no liquefaction occurred, although the growth was considerable in both serum and agar tubes. Upon gelatin, however, no liquefaction occurred, even when a decalcified slice was hung for seven days in the liquefied medium. The control tubes were unaltered and showed no growth. From this it follows that liquefaction of dentine may be caused by the digestive enzyme produced by certain bacteria, and that an organism, although having the power to liquefy gelatin in the culture tube, does not therefore possess the power of liquefying dentine. So far as I can find, no other observers have made this extremely simple experiment, which is of considerable importance in the pathology of dental caries.

---

[1] Journal of the British Dental Association, March, 1899.

In making cultivations from the deep layers of carious dentine I have so often met with one organism, a bacillus often in pure culture, that its relation to the carious process cannot be entirely accidental, and, as several of its characteristics fit in with the process of decay, it is important to describe it in this connection. Ordinary laboratory media are adapted to its growth, as of all those organisms to which I have referred in the foregoing remarks.

The method used in its isolation was as follows: Extracted carious teeth were immediately taken to the laboratory, and the surface of the dentine seared with hot instruments in the method always adopted in making post-mortem cultures from infected animals. The outer layer of caries was then removed with sterilized instruments, the surface again seared, and another layer removed with a fresh instrument, and cultures on agar and coverslip preparations made. The direct preparations showed short, thickish bacilli, often in almost pure culture, which stain deeply by Gram, as well as some finer bacilli, cocci, and commas, the cocci being arranged as diplococci. Broth cultivations were also made at the same time.

In thirty-six to forty-eight hours a slight growth of minute, round, gray-white translucent colonies developed upon the surface of the agar. Coverslip preparations made from these colonies show a mixed growth of streptococcus brevis and the bacillus I am about to describe. In the broth tubes the growth is mainly streptococci, although a few of the bacilli may also be found.

The cultures were then plated and a pure colony of the bacilli picked out and streaked on to the surface of agar. From the situation in which this organism is found, Dr. Perry, of Guy's Hospital, has suggested to me the name of B. necrodentalis.

The organisms were isolated in this manner from twenty cases of caries, the organisms having the same general characters as follows:

BACILLUS NECRODENTALIS.

*Morphology.*—Short bacilli three-fourths centimetre wide, one to five centimetres long, often associated in pairs and sometimes in chains; the ends of the bacilli are rounded or square. The bacilli tend to involute rather rapidly and produce swollen and contorted masses, not unlike the involution forms of the streptococcus. In broth the forms are extremely like cocci, especially in the hanging drop owing to the shortness of the elements; this is particularly well marked when in the form of chains (strepto-bacilli). The bacilli are slightly motile on aërobic cultures, but more so on anaërobic ones.

*Staining Reactions.*—Stains by Gram's method and by ordinary aniline dyes, with difficulty by methylene-blue. Many lightly stained involution forms are to be seen on three-day-old cultures on agar. No partial staining or polar staining has been noticed. Spore formation has so far not been observed.

*Biological Characters.*—Anaërobic, facultative, aërobic, non-liquefying, slightly motile bacillus.

(1) *Gelatin Plates.*—In three to five days minute pin-point colonies may at times be seen, very similar to streptococcus colonies.

(2) *Gelatin Streak.*—Slight dotted growth of minute colonies along the streak in three days; no liquefaction of gelatin occurs.

(3) *Gelatin Stab.*—Three days, slight dotted growth to depths of stab, no marked surface growth; no liquefaction in three weeks.

(4) *Gelatin Shake.*—Five days, cloud of minute whitish-gray colonies; no liquefaction of medium.

(5) *Broth.*—Twenty-four hours, slight general turbidity with precipitate, which may be stringy on giving the tube a circular shake. The turbidity increases very little, but the precipitate increases considerably in forty-eight hours.

(6) *Potato.*—Forty-eight hours, very slight shining appearance. Coverslip shows presence of bacilli, with considerable involution.

(7) *Litmus Milk.*—Twenty-four hours, no change in medium. Forty-eight hours, solid clot, with the lower portion decolorized, the top showing a strongly acid reaction. The clot does not become redissolved.

(8) *Agar Streak.*—Twenty-four to forty-eight hours, slight grayish translucent dotted growth, which never develops very much more. Typical short thick bacilli.

(9) *Blood-Serum.*—Very little growth, flat, almost invisible.

*Indol.*—None in seven days.

*Gas.*—None observed.

*Anaërobiosis.*—Grows best when oxygen is excluded in ordinary anaërobic tubes, and especially on glucose media. Scarcely at all on blood-serum.

On agar the colonies are: (1) Macroscopically—gray-white, round and regular, or slightly wavy, with a slight central prominence. (2) Microscopically—brownish, with slight central dot, regular edge, and faintly granular.

In almost any case (sixteen out of twenty), I have also obtained the streptococcus brevis, and as both these organisms are anaërobic, B. necrodentalis especially, and from the power they have of acid production, they appear to be largely concerned in the decalcification of the matrix prior to its disintegration by other organisms. The other forms noticed have not so far been isolated upon the ordinary media. The organism, as will be seen from the photograph of the agar culture, is remarkably pleomorphic, even before involution forms have formed.

There is one other organism which I have found constantly associated with others in dental caries (surface), and in two out of the twenty cases in which the deeper layers were examined, and in which B. necrodentalis occurred. It belongs to a species hitherto undescribed in the mouth; in fact, Miller says, with the exception of "cladothrix and beggiatoa" (sewage organisms), all the most common species of bacteria have their representatives in the mouth. Now, the organism I am about to describe to you appears to me to belong to the first of the above two groups,—*i.e.*, cladothricæ.

The cladothricæ are a curious class of organisms which occur in sewage, and, unfortunately, commonly in waters, especially when there is any putrefaction of any animal or vegetable matter. According to Zopf, "*Spherical, rod-shaped, filamentous, or spiral forms, the filaments show pseudo-branching spore formation not known.*" According to other observers, the branching is not pseudo, but actual, and there appears some reason for supposing that both forms actually exist,—*i.e.*, real and pseudo dichotomy. Cladothrix dichotoma certainly show pseudo-branching; the whole genus has, as a characteristic in common with crenothrix, beggiatoa, and phragmidothrix, a remarkable pleomorphism, and as they form threads, isolated and branched, unbalanced minds have decided, without experiment, that the class as a whole is the author, progeny, and actuality of all existing organisms; but you will easily see, from what I have said concerning the heads of the so-called leptothrix racemosa (crenothrix), bacteria, as stock-keepers say, "breed true," and that when the proper precautions are observed, the organisms may be watched through their various stages of development, and that any phrase development ultimately undergoes all the typical stages till it returns to the same form again—an insurmountable difficulty to those who suggest that new organisms, carefully described, are merely phase forms of the hypomycetes.

A scraping from the enamel of the teeth, and examined in the hanging drop, generally shows a large variety of threads of many sorts, called by older observers by the inclusive term, "leptothrix" (*i.e.,* threads). Upon staining such a mass by Gram I have often found a curious branching organism, the branches for the most part being the pseudo-branching of cladothrix, but when stained the gap between thread and thread is filled up. So far I have only found this organism in twenty mouths, but as they varied from the pathological hyperæmia of gingivitis to the normal mouth of an infant of six, the selection is fairly representative. The organism is often the only one which retains Gram's stain after the decolorization by chloroform and alcohol alternately. By the ordinary Gram method (alcohol alone used for decoloring) other organisms are stained. This remarkable organism, whose pleomorphism is unbounded, can be obtained in pure cultivation. I suggest for it the name of cladothrix buccalis; it is as follows:

CLADOTHRIX BUCCALIS (GOADBY).

*Morphology.*—Rod-shaped, filamentous forms, with general appearance of branching, except in the hanging drop. The threads are somewhat slender, and the ends are often club-shaped. The threads often appear articulated, like a strepto-bacillus, similar to cladothrix dichotoma (Cohn). The branching does not, as a rule, show such a dichotamous nature as is seen in the other described forms of cladothrix, the branches appearing more at right angles to the main stem, especially in agar cultivations. On potato, an unparalleled pleomorphism occurs, and all varieties of morphology may be seen, spirillæ, threads, bacilli, cocci. The development of the threads from the cocci may be seen. The clubbed ends take the stain deeply.

*Staining Reactions.*—Stains by Gram and by ordinary aniline dyes; the threads often have semi-stained portions. The clubs take a deep stain.

*Biological Characters:* (1) *Gelatin.*—Little growth, no liquefaction. Fourteen days, liquefaction at surface of stab.

(2) *Agar.*—Forty-eight hours: small, hard, round, raised, transparent, or slightly cloudy colonies, about the size of a pin's head. Three days: raised, truncated cones, whitish brown, and in the older colonies a slight crack appears across the top of the cone. The colonies eventually cause a slight depression in the agar. They are extremely difficult to remove with the platinum needle, and must be removed entire. The agar is colored a dirty brown. These colonies are extremely characteristic. The coloration is not constant.

(3) *Blood-Serum.*—Similar to agar; no liquefaction takes place.

(4) *Broth.*—Very little growth, slight flaky precipitate.

(5) *Potato.*—Yellowish or orange-brown hard flat layer; the top of the potato is often covered with round chalk-white colonies about the size of small shot.

(6) *Litmus Milk.*—No acid reaction, no clotting.

A curious earthy sour smell is given off from the cultivations, which reminds one of a damp, musty cellar.

RÉSUMÉ.

In summing up the foregoing remarks we find the question falls under two divisions:

(1) The relation of certain organisms to the destruction of tooth tissue. (2) The difficulties in separating true species from morphological forms, and, I may add, the question of occurrence of the hypomycetes in the mouth.

To take the latter first. The fruitful heads and other phenomena of Vincentini's L. racemosa, as observed by myself in the coverslip plates, agree in many respects with the crenothrix Kühnina of Rabenhorst. (1) The morphological variety of its arthrospores. (2) The small transverse sections of Leon Williams seem to me to be small zooglœa masses, especially as in the broth hanging drop these masses developed threads in the manner described by Rabenhorst and Zopf.[1]

These little tufts of threads are often to be seen in preparations stained direct from the mouth. Under these circumstances crenothrix buccalis is a better term than leptothrix racemosa. The cladothrix buccalis (Goadby), which I have described to you, differs in many respects from the cladothrix dichotoma of Cohn. As no

---

[1] Leon Williams has since demonstrated to me that these transverse sections are the ends of the "fruitful heads" seen *end on.*

sulphur grains were observed, the question of beggiatoa is eliminated. The phragmidothricæ have, I believe, so far only been found in sea-water; however, phragmidothrix multiseptica is not at all unlike the crenothrix we have discussed. The cladothrix buccalis has such remarkable pleomorphism, especially in potato cultures, and corresponds so closely with Zopf's nomenclature, that I think we may leave the matter for the present.

In dental caries a number of organisms have been described which color the culture media a deep brown. I find the potato bacilli, B. mesentericus, ruber, vulgatus, fuscus, commonly in the mouth; they are among those that discolor the medium. Arkövy's B. gangrænæ pulpæ produces discoloration, but has a distinct and unpleasant smell, like bad cheese; it differs from B. necrodentalis (Goadby) in this respect as well as in the liquefaction of blood-serum and gelatin. Miller's B. fuscus colors agar brown, but no further details are given. Jung describes an organism (coccus or bacillus not stated), which, from its reaction, appears to be B. mesentericus. Martin Freund gives three chromogenic, but the pigment differs in many respects from those organisms I have described. So far I find no other organism in any way resembling the B. necrodentalis.

The liquefying organisms I have isolated appear not to correspond with those isolated by Vignal.

In conclusion, I maintain that in dental caries (1) the liquefying organisms are mostly aërobic; (2) the acid-producers are largely anaërobic; (3) that liquefaction of gelatin does not imply digestion of dentine, but liquefaction of blood-serum may; and, finally, that the demonstration of cladothrix in the mouth does not imply that all the other organisms of the lower class of scherzomycetes found in the mouth are simply phase forms of the hypomycetes.

Since the above paper was written I have made a considerable number of observations on the genus cladothrix.

The cladothrix which I have isolated from the mouth, and discussed at some length above, differs from the following varieties in its biological and other reactions:

C. dichotoma of Cohn; C. nivea; and from the cladothrix given to me by Leon Williams as well as several other species, cultures of which have been sent me by Dr. MacConkey, of the Royal Commission on Sewage.

I have also isolated C. dichotoma from several mouths and throats as well as the C. buccalis.

The culture given me by Leon Williams most resembles the cladothrix described above, but, nevertheless, differs from it in many respects.

I hope shortly to publish more fully the details of these experiments.—*Journal British Dental Association.*

